# A case of high-grade adenosquamous carcinoma of the breast: case report and literature review

**DOI:** 10.3389/fonc.2025.1548036

**Published:** 2025-03-04

**Authors:** Xiaoxiao Xing, Junyi Li, Yangyang Fan, Yun Wang, Yue Wang, Daixiang Liao, Shiyun Zhang

**Affiliations:** ^1^ Department of Surgery, Guang’anmen Hospital of China Academy of Chinese Medical Sciences, Beijing, China; ^2^ Department of Radiology, Guang’anmen Hospital of China Academy of Chinese Medical Sciences, Beijing, China; ^3^ Department of Pathology, Guang’anmen Hospital of China Academy of Chinese Medical Sciences, Beijing, China

**Keywords:** high-grade adenosquamous carcinoma, metaplastic breast cancer, case report, breast cancer surgery, adjuvant chemotherapy

## Abstract

High-grade adenosquamous carcinoma (HGASC) is a rare and aggressive subtype of metaplastic breast cancer (MpBC). This article reports a case of HGASC (pT2N0M0 Stage IIA) in a 43-year-old female and reviews the relevant literature, with a specific focus on distinguishing HGASC from other MpBC subtypes, particularly low-grade adenosquamous carcinoma (LGASC). The patient underwent a skin-sparing mastectomy with abdominal rectus myocutaneous flap reconstruction. Histopathology confirmed HGASC with metaplastic features. Postoperative adjuvant chemotherapy with capecitabine was administered. The case highlights the unique clinical, imaging, and pathological characteristics of HGASC, its therapeutic challenges, and the need for individualized treatment strategies. A five-month follow-up showed no signs of recurrence or metastasis.

## Background

Metaplastic breast cancer (MpBC) is a rare and aggressive malignant tumor, accounting for approximately 0.2%–1% of all breast cancers (1). In 2019, the World Health Organization (WHO) revised the histological classification of MpBC based on the stromal and epithelial components of the tumor. The current subtypes include low-grade adenosquamous carcinoma, fibromatosis-like metaplastic carcinoma, squamous cell carcinoma, spindle cell carcinoma, metaplastic carcinoma with heterologous mesenchymal differentiation (such as chondroid, rhabdomyoid, or osseous), and mixed-type metaplastic carcinoma (2). Among these, low-grade adenosquamous carcinoma (LGASC) is indolent and high-grade adenosquamous carcinoma (HGASC) of the breast is an extremely rare subtype, characterized by the simultaneous presence of adenocarcinoma and squamous cell carcinoma components with high invasiveness, accounting for approximately 0.05%–0.1% of all breast cancers (3). Clinically, HGASC is characterized by large tumor size, rapid growth, high invasiveness, and low axillary lymph node metastasis rate (4). This case report aims to shed light on the clinical presentation, imaging, pathology, and treatment of HGASC, emphasizing its distinction from other MpBC subtypes.

## Case presentation

A 43-year-old female presented with a two-month history of a palpable mass in the right breast. Approximately two months ago, the patient noticed localized pain in the right breast and palpated a hard mass. There was no tenderness upon palpation, no fever, no local skin redness, no nipple discharge, or nipple retraction. Mammography at a local hospital showed a Breast Imaging - Reporting and Data System (BI-RADS) 4B lesion in the right breast. On July 3, 2024, the patient visited our hospital for further evaluation. A breast biopsy was performed, revealing irregular small glands and small nest-like clusters of cells in the fibrous and sclerotic stroma. The cells showed atypia, and scattered mitotic figures were observed. Metaplastic carcinoma with sclerotic changes could not be ruled out. The biopsy was localized, and excision followed by comprehensive evaluation was recommended. The immunohistochemical results were: Estrogen Receptor (ER)(-), Progesterone Receptor (PR)(-), Human Epidermal Growth Factor Receptor-2 (HER-2)(-), Antigen Ki - 67 (Ki-67) (+10-20%).

Upon admission, the patient had a palpable right breast mass with associated pain, but there was no nipple discharge or fluid leakage. The patient reported premenstrual breast pain, which was linked to emotional stress. The patient had a history of hypertension for 7 years, well-controlled with medication. On physical examination, the breasts were symmetrical, with no nipple inversion or signs of peau d’orange or dimpling. A hard, tender mass of approximately 2×2 cm in size was palpated at the 12 o’clock position of the right breast. The mass had an irregular surface and poor mobility. There was no nipple discharge or arm edema. No significant mass was palpated in the left breast, and no axillary lymphadenopathy was observed bilaterally.

**Figure 1:**
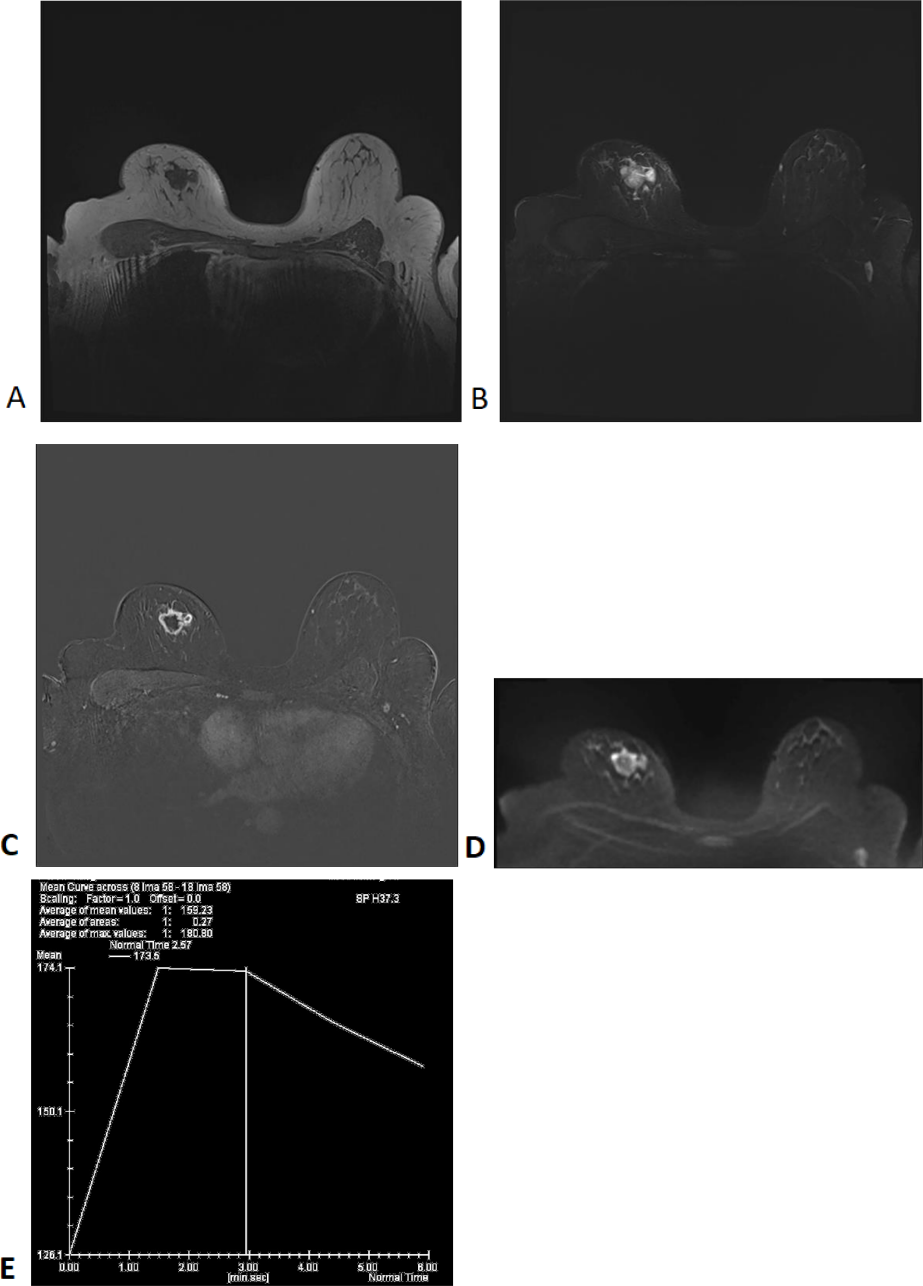
MRI Findings: T1WI **(A)**, T2WI **(B)**, T1WI+Contrast **(C)**, DWI **(D)**, DCE-Curve **(E)**.

Auxiliary Examinations: Enhanced breast MRI (**Figure 1**) showed a lobulated mass in the right breast with a size of approximately 22×27×21 mm. T1-weighted imaging (T1WI) showed low signal intensity, while T2-weighted imaging (T2WI) demonstrated slightly increased signal intensity, with cystic changes in the central region. Diffusion-weighted imaging (DWI) revealed a thick-walled mass with high signal intensity, irregular shape, and low signal intensity in the central area. The mass was irregularly enhanced during the early phase of contrast injection, with spiculated margins. The central necrotic area showed no enhancement. The dynamic contrast-enhanced curve(DCE-Curve) showed a washout pattern, consistent with squamous metaplasia but not definitive.

Considering the patient’s age and preferences, she underwent a skin-sparing mastectomy with abdominal rectus myocutaneous flap reconstruction. During surgery, sentinel lymph node biopsy was performed, and intraoperative frozen section pathology showed no evidence of metastatic carcinoma. The breast tissue was excised, extending to the surface of the pectoralis major fascia. The excised specimen measured approximately 12×10 cm, with the remaining breast duct removed. Frozen section pathology confirmed negative margins with no evidence of metastatic carcinoma.

**Figure 2:**
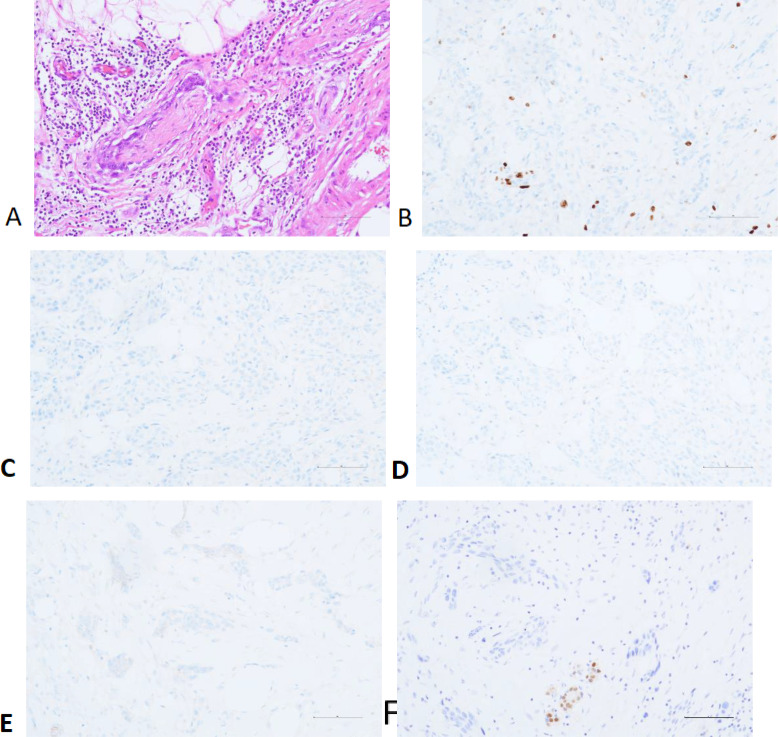
Perineural Invasion **(A)** and Immunohistochemistry: Ki-67 **(B)**, ER **(C)**, PR **(D)**, HER-2 **(E)** and AR **(F)**.

Postoperative Pathological Results: The right breast and partial skin tissue excision specimen showed invasive carcinoma with extensive necrosis. The cancer cells were arranged in nest-like patterns, with some spindle-shaped cells and others showing epithelial-like morphology and eosinophilic cytoplasm. The diagnosis was metaplastic carcinoma (high-grade adenosquamous carcinoma) (pT2N0M0 Stage IIA), measuring 2.3×1.8×1.7 cm, with evidence of nerve invasion but no vascular invasion. The skin, base, and surgical margins showed no cancer involvement. Immunohistochemistry results indicated: Ki-67 (+30-40%), ER(-), PR (+<1%, weak staining), HER-2 (1+), Androgen Receptor (AR)(-) (**Figure 2**).

The patient recovered well postoperatively and, after discussion, underwent six cycles of adjuvant capecitabine therapy. Regular follow-up examinations were conducted, and at the five-month follow-up, there was no evidence of recurrence or metastasis.

## Discussion

Metaplastic breast carcinoma (MpBC) comprises various histological subtypes, among which high-grade adenosquamous carcinoma (HGASC) represents a distinct entity with unique characteristics. Several studies have demonstrated significant differences between HGASC and other MpBC subtypes, particularly low-grade adenosquamous carcinoma (LGASC), across multiple key aspects (5).

As a rare malignant breast tumor, the relationship between the clinicopathological features, treatment approaches, and clinical outcomes of MpBC remains insufficiently studied (6). In clinical practice, HGASC typically presents on MRI as an irregular mass with spiculated margins, indistinct boundaries, central necrosis, heterogeneous enhancement, and a rapid washout pattern on dynamic contrast-enhanced imaging. A multicenter imaging study analyzing MRI scans of 150 MpBC patients found that these typical features were observed in up to 85% of HGASC cases. In contrast, LGASC often appears as a smaller, well-defined lesion with relatively mild imaging features. Due to its well-demarcated borders and lower enhancement intensity, LGASC is frequently misdiagnosed as a benign sclerotic lesion, typically displaying a more homogeneous enhancement pattern (7).

Clinically, HGASC exhibits greater aggressiveness. A Study has shown (8) that HGASC tumors grow rapidly, and patients often present with larger tumor sizes at diagnosis. Some cases may exhibit breast pain, peau d’orange changes, and a higher likelihood of axillary lymph node and distant metastasis. Conversely, LGASC exhibits slower growth, smaller tumor sizes, and a lower risk of metastasis, with most patients presenting only with a painless breast mass (9).

From a pathological perspective, HGASC is characterized by poor differentiation, marked cellular atypia, and a high mitotic rate, which correlates with its aggressive behavior and poorer prognosis (10). In contrast, LGASC demonstrates better differentiation, lower cellular atypia, and reduced mitotic activity (9).

Immunohistochemically, Ki-67 serves as a proliferation-associated antigen, with high Ki-67 expression levels often linked to increased tumor cell proliferative activity (11). A Study has suggested that although HGASC exhibits high aggressiveness, intratumoral heterogeneity may exist, with tumor cells in different regions displaying varied biological characteristics, including Ki-67 expression levels. This heterogeneity may impact treatment response and prognosis (12). Consequently, although HGASC generally exhibits high Ki-67 expression, its prognosis may not necessarily be as poor as other high-grade tumors (13). In contrast, LGASC typically exhibits lower Ki-67 expression levels, generally ranging from 5% to 15% (14).

Regarding treatment, most MpBC subtypes are classified as undifferentiated triple-negative breast cancer (TNBC), which is insensitive to endocrine therapy and targeted therapy, resulting in a poorer prognosis (15). Currently, surgery remains one of the primary treatment options for highly aggressive MpBC subtypes such as HGASC (16). Studies have found no significant difference in survival rates between mastectomy and breast-conserving surgery, though the latter is associated with a higher local recurrence rate (17). Therefore, in clinical practice, HGASC patients often require wider surgical excision. For tumors with larger sizes or surrounding tissue invasion, radical surgery is often necessary to ensure complete tumor resection (18). In contrast, LGASC, as an indolent subtype of metaplastic breast carcinoma, rarely metastasizes. Consequently, breast-conserving surgery with negative margins is the preferred approach, while adjuvant radiotherapy, endocrine therapy, or chemotherapy is generally unnecessary (14).

In terms of adjuvant therapy, HGASC patients exhibit significantly lower survival rates than other breast cancer subtypes, particularly those with tumor diameters >2 cm, lymph node metastasis, and ER-/PR-negative status, which are associated with higher recurrence risks and shorter disease-free survival (19). Studies suggest that due to its high malignancy, HGASC exhibits relatively low sensitivity to conventional chemotherapy agents such as anthracyclines and taxanes, often requiring higher doses or combination regimens (13). Given its biological similarities to TNBC, clinicians often refer to TNBC treatment protocols, selectively incorporating capecitabine. While no specific molecular targets have been identified, future immunohistochemical analyses indicating high PD-L1 expression or increased tumor mutational burden may support the use of PD-1/PD-L1 inhibitors in combination with chemotherapy to improve treatment outcomes in HGASC (20).

In conclusion, the high aggressiveness and poor chemotherapeutic response of HGASC make it one of the more challenging breast cancer subtypes in terms of prognosis. Further large-scale studies are needed to optimize treatment strategies. Based on current evidence, surgical intervention and adjuvant therapy may provide survival benefits, but the specific efficacy and optimal treatment protocols require further validation and investigation.

## Patient perspective

Upon being diagnosed with high-grade adenosquamous carcinoma (HGASC), the patient initially experienced distress and concern due to the aggressive nature of the disease and the relatively limited treatment options. The rapid tumor growth caused both physical and emotional distress, significantly impacting the patient’s well-being. Throughout the treatment process, the patient underwent a comprehensive evaluation and a multidisciplinary treatment plan. Following surgical resection, the patient reported a sense of relief upon knowing that the primary tumor had been removed. However, anxiety persisted regarding the potential for recurrence and the limited effectiveness of conventional chemotherapy. During postoperative chemotherapy, the patient experienced side effects such as fatigue and numbness in the fingers. Nevertheless, she remained optimistic, adhered to the treatment regimen, and attended regular follow-up appointments. The patient expressed appreciation for the overall treatment process and gratitude toward the medical team for their efforts in tailoring a personalized treatment plan. Furthermore, she expressed a strong willingness to contribute to future research endeavors, hoping that her experience could help improve treatment strategies for others facing similar diagnoses.
